# An unfortunate natural experiment in learning how to provide services to those in need: The case of Ukrainian war refugees with disabilities in Warsaw and Bucharest

**DOI:** 10.1371/journal.pone.0311331

**Published:** 2025-05-22

**Authors:** Monika Nowicka, Alexandra Deliu, Bogdan Voicu, Magdalena Szarota

**Affiliations:** 1 Institute of Sociology, Civitas University, Warsaw, Poland; 2 Research Institute for Quality of Life, Romanian Academy, Bucharest, Romania; 3 Sociology, Lucian Blaga University of Sibiu, Sibiu, Romania; 4 National University of Science and Technology “Politehnica”, Bucharest, Romania; 5 Department of Sociology, Lancaster University, Lancaster, United Kingdom; Wrocław University of Science and Technology, POLAND

## Abstract

When helping others, experience becomes important, especially in circumstances that involve interacting with different cultures—such as providing services to refugees. When disability is added to refugee status, multiple types of experience become necessary, with cross-sector collaboration serving as a valuable asset. Thus, in our approach, we do not consider the status of being a migrant and the status of being a person with disabilities separately, as that would be contrary to the lived experience of simultaneously being a refugee and person with disabilities. This paper explores the impact of the Russian invasion of Ukraine on the capacity of the Polish and Romanian organizations providing services to Ukrainian refugees in Warsaw and Bucharest, with a particular focus on disabled refugees. Based on 41 interviews with service providers and grass-roots organizations, we find that this unfortunate event functioned as a natural laboratory for practicing, acquiring, and enhancing skills in multiple domains, leading to increased personal and institutional expertise. We examine the differences between Warsaw and Bucharest, with Warsaw having more experience in dealing with incoming flows of immigrants, while Bucharest is a relative newcomer in this respect. Additionally, we consider the distinction between public providers (public administration) and non-governmental organizations, observing the upscaling of the latter. Implications for policy are discussed within the framework of curtailing civic society under the illiberal wave.

## Introduction

Lessons learned by civil society and public service providers from dramatic events can significantly improve both society at large and civil society in particular by enhancing procedures, fostering cross-sectoral cooperation, and preemptively addressing crises [1–3]. In this paper, we ask what lessons can be drawn from the *ad hoc*—or “spontaneous”—enthusiastic phases of helping Ukrainian refugees in Poland and Romania. We focus specifically on the encounters of service providers with war refugees with disabilities, treating this negative life event as a natural experiment that compels societies and organizations to respond quickly and efficiently.

In historical terms, the Russian invasion of Ukraine was not entirely unexpected, given that Russian forces have repeatedly occupied the region, both during the tsarist era and the Soviet period. Prior acts of aggression in the Black Sea area (e.g., against Moldova and Georgia) as well as the 2014 invasion of Crimea, preceded the full-scale invasion of Ukraine in March 2022. However, the scale of this invasion was unprecedented in post-World War II Europe, eliciting a strong emotional response across the continent. This reaction influenced both citizens and policymakers, particularly—but not exclusively—in countries bordering Ukraine [4–7].

At the same time, despite people with disabilities in Western culture having high deservingness [8,9], particularly when disabilities are based on medical evidence [10], post-communist European societies have experienced surges in dissatisfaction from people with disabilities regarding their treatment [11,12]. Neglecting social policy costs in favor of economic growth and maintaining barriers in accessing social services by the beneficiaries have fueled such reactions [13]. This situation persists in societies where special needs remain highly stigmatized [14] and where little attention is paid to disability beyond the medical model [15]. In times of recession, when state retraction is anticipated, the professionalization of non-governmental service providers is expected to prevail, thereby redressing the growing inequalities in accessing care [16], yet reports suggest that many such organizations are still in their “infancy” phase [17].

Sudden influxes of refugees typically strain existing healthcare and welfare systems, as demonstrated by cases across Europe [18–20] or as forewarned by healthcare specialists in the early days of conflicts [21]. Although international regulations mandate assistance for disabled refugees [22], implementation is often inconsistent, with challenges becoming especially acute in times of crisis. Even well-established welfare regimes such as Australia have struggled to accommodate refugees with disabilities due to differing interpretations of rights and to defining their disability at multiple points of entry into the protection system [23]. Generally, these difficulties arise from a lack of coordination between public sector administrative systems devoted to healthcare and those addressing disability [24].

Consequently, we apply the concept of intersectionality in our analysis. It was initially introduced in relation to gender and race by [25,26] to argue that different groups of women experience discrimination differently and to draw attention to the marginalization of black women’s experiences. Intersectionality has evolved to encompass numerous variables and is now widely used in diversity studies [27]. Within this framework of intersectionality, we argue for the importance of paying attention to how different, overlapping statuses or attributes position individuals in spaces of intersection that cannot be fully understood when these attributes are considered separately. Our study contributes to the still-developing literature that jointly examines migration and disability [28–35], adding the dimension of post-communist residual welfare regimes, which are increasingly threatened by illiberal shifts. This addition is significant, as less-developed care regimes or those with less benevolent attitudes have been identified as environments where disabled migrants are typically overlooked and, thus, particularly vulnerable [34].

To summarize, while people with disabilities are often depicted as lacking agency and in need of charity, adding refugee status makes them even more vulnerable. Furthermore, weaker welfare provisions and an illiberal political climate exacerbate this intersectional disadvantage. This context raises important questions about how service provision evolves under such conditions. Our research offers a valuable opportunity to examine the evolution of service providers in Poland and Romania—two EU member states bordering Ukraine, both of which have received significant numbers of refugees, share a post-communist history, and harbor strong anti-Russian sentiments while having less developed welfare systems.

This study is based on 41 interviews conducted in Warsaw and Bucharest in 2023 with service providers and regulators in the field of disabled refugee assistance, including state agencies and non-governmental organizations (NGOs). We analyze the interviews to understand how these providers have evolved over time and whether this crisis has enhanced their ability to help refugees, immigrants, and the native populations. Our focus is on scaling up service provision, developing and learning ways of doing; we explore whether and how they develop intra- and cross-sector cooperation, within their sector and beyond their sector; and assess whether the experience has translated into newly acquired or improved competences. While previous studies have stressed the general impact of the war on NGOs in the region [36,37], we go deeper, considering cross-sectorial effects, intersectionality, and long-lasting changes. We also compare public service providers in two capital cities. Since negative societal events often create windows of opportunity [2,38,39], we expect increases not only in terms of internal practices and immediate expertise, as observed by other scholars [37,40], but also in terms of cross-sectorial cooperation and the capacity to extend activity into other areas.

In this analysis, we present a four-fold framework. We focus on the local contexts in which organizations dedicated to assisting migrants are active while also examining service providers for individuals with disabilities. Additionally, we explore concepts of deservingness and investigate the challenges that NGOs face amidst the current illiberal climate. All these elements contribute to the background of our story. A brief section on “hypotheses” concludes this background discussion, followed by a detailed methodology section that explains our empirical sources, which are then extensively analyzed in the findings section. The paper concludes with an exploration of the dimensions of organizational change and upscaling that migration and disability NGOs and public service providers have experienced and highlights their implication on organizational structures, policy, and research.

## Literature review

### Migration/refugee-centered organizations: Aims and approaches to disability

Across the world, service providers working with immigrants—both public and NGO-run—are often reported to avoid serving people with disabilities [34,41,42]. This reluctance stems from prejudice, difficulties in addressing multiple vulnerabilities, and a lack of coordination between different systems, particularly public service providers [23,24,43–45].

Both Romania and Poland have short histories of receiving migrants, as they have historically been better known for exporting migrants. However, in recent years, the number of economic immigrants in both countries has been increasing [46,47].

In both countries, NGOs serve as the primary providers of assistance and services for voluntary and non-voluntary migrants [48,49]. These organizations focus on activities that help refugees and migrants adapt to life in a new country, providing support in cultural, economic, and social areas, either complementing the actions of public institutions or replacing them when state support is lacking [50]. Although NGOs are central to assisting immigrants, their efforts extend beyond this population to address broader societal needs in receiving countries [48,51].

The scope of NGO activities ranges from short-term aid—such as emergency relief, legal assistance, and educational programs—to long-term involvement in advocacy and advising on policy development [52,53]. However, NGOs assisting migrants and refugees rarely provide support specifically for people with disabilities, as migrants are typically expected to be able to work. Even when migrants have disabilities, they often remain invisible within the system [29,54], despite being a significant part of migratory flows [31]. At the same time, the intersection of migration and disability is particularly relevant for individuals fleeing at-risk areas.

Even during refugee flows, the level of support provided to refugees with disabilities depends on various factors, such as the capacity of reception centers. For example, in the Greek islands, which are known for their struggle with significant flows of people seeking international protection, a large number of refugees can render people with disabilities invisible [55]. In contrast, Austria, where refugee numbers are significantly lower, has established procedures for assisting refugees with disabilities [56]. Another major challenge in providing aid is the unclear legal status of these individuals, which can restrict access to essential services [56], particularly health care [29]. Other factors obstructing effective and efficient support include a lack of reliable data and the absence of standardized methods for assessing vulnerability [57].

### Disability-centered organizations: Aims and approaches to migration

Disability researchers have emphasized the critical role of disability organizations in post-socialist regions, especially given the scarcity of state-provided services for disabled persons [58,59]. These organizations have therefore filled significant gaps in the provision of essential medical, welfare, and societal support systems that have been neglected in the transition to capitalist economies in countries like Romania and Poland [60,61]. This reliance on disability organizations has arisen due to the state’s failure to provide adequate services and the high costs imposed on disabled individuals, rendering such services financially inaccessible in heavily privatized sectors.

This situation has disproportionately affected disabled people in post-socialist regions, who, for a variety of reasons, typically belong to the most economically disadvantaged segments of society. Consequently, disability organizations have traditionally focused their efforts on impairment-specific assistance rather than adopting a more inclusive, cross-impairment approach [17,58,59]. This approach has been shaped in part by state funding mechanisms, which prioritize objectives such as facilitating employment for disabled individuals and social integration through work-related programs. Disability studies scholars have noted the pragmatic approaches these organizations employ to secure resources for their vital activities, compensating for state inadequacies.

The adoption of the Convention on the Rights of Persons with Disabilities (CRPD) has led some disability organizations—both large and small—to shift their focus toward aligning their objectives with the values outlined in the CRPD. These organizations have begun advocating for a transition away from medical and charity models of disability toward a more comprehensive human-rights-based model—a shift that aimed to address disability discrimination in a systematic way, acknowledging its intersectional and holistic nature [61,62].

Before the arrival of Ukrainian war refugees with disabilities (UWRwD) in Poland, Polish disabled-focused NGOs did not support migrants and refugees. This is reflected in the sparse literature on health-related issues in the context of migration and refuge. Previous research has primarily focused on access to public health services and social security issues [63,64].

The intersection between disability and migration has received minimal attention in the literature on immigration in both Romania and Poland [65–68] and, to the best of our knowledge, has not been comprehensively analyzed. Prior to the current Ukrainian refugee crisis, reports on social service provision in these countries have largely overlooked immigration-related concerns [e.g., 69,70,71]. However, more recent reports present a different picture, reflecting the increasing involvement of service providers in addressing both disability and refugee-related challenges at the same time [e.g., 37,72]. The absence of migrants in past reports on disability is unsurprising, as public service providers and NGOs assisting people with disabilities in various parts of the world have generally had little interaction with disabled migrants.[e.g., 24,37,72,73].

### Experiences shaping the approaches toward aiding refugees

Public actors—including administrative bodies at various levels (local, regional, central)—play a crucial role in providing services to those in need, including refugees. Their capacity to respond to the needs of potential beneficiaries depends not only on the competencies of their members but also on network cooperation across different types of actors (public, civil society organizations) [74]. Additionally, both public service providers and civil society organizations face interconnected challenges in their efforts [75].

Civil society organizations provide services to those excluded by public policies or facing immigration restrictions [76]. Their role in integrating refugees and asylum seekers has been extensively studied [77,78]. The effectiveness of their activities depends on communication, cooperation, and coordination between service providers [79] and is influenced by the attitudes and actions of local authorities [80]. These organizations must comply with regulations governing forced migration and the integration of displaced populations, which often reinforce existing policies or discriminatory practices [76,81].

NGOs serving refugees and asylum seekers generally fall into two categories: those specializing in service provision and those primarily engaged in advocacy. Additionally, NGOs can be either majority- or migrant-led organizations, depending on their membership composition [82]. Ultimately, these organizations must balance state policies of exclusion with their commitment to human rights and refugee support paying attention to basic human rights [83]. This tension between care and control becomes even more pronounced when the (limited) funds available to NGOs are distributed/managed by public authorities, decreasing the propensity of NGOs to advocate or uphold stances against their (main) funders [82,84–86].

The duality between perceiving refugees as victims/recipients of help, on the one hand, and as individuals with their own agency, on the other, seems to shape the attitudes of NGOs as well as their ways of engaging with their clients/beneficiaries, which are closely linked to the characteristics of the organizations. For example, (migrant-led) refugee community organizations contribute to the empowerment of refugees, while NGOs, as a result of their more formal ways of organizing and delivering services, instead tend to see refugees as recipients of help [79]. When refugees are seen as victims, this actually contributes to making them passive, by stripping them of their agency [83].

Existing scholarship has highlighted several key issues [80]: the spaces of interaction between service providers and beneficiaries as well as the role of refugees and asylum seekers as an empowerment mechanism [82,87]; the bureaucratic obstacles and need for adequate training among frontline social workers [83]; and the challenges of operating in hostile environments, where public attitudes toward immigration and forced migration are negative, particularly in contexts where governments impose restrictive policies [88].

### The framework for curtailing civic society amid the illiberal wave

In the 1990s and 2000s, Central and Eastern Europe experienced low levels of civic participation, a lingering effect of their totalitarian pasts [89]. Efforts to encourage voluntary associations were not always clearly defined [90]. While recent literature has reported an increase in participation [91–93], progress has been slow [94,95], coinciding with over a decade of rising populism, illiberal governance [96,97], and aggression toward NGOs [36,98–100].

Paradoxically or not, at the same time, post-communist societies, including Poland and Romania, have increasingly recognized the critical role of civic organizations in supplementing state welfare services, given their greater flexibility and adaptability in terms of providing tailored programs [16,17,58,59]. This has resulted in a complex landscape marked by two opposite trends: a growing reliance on civil society due to its social function—particularly in response to crises such as war—and, simultaneously, the emotional reactions towards Russian aggression; and second, a contrary backlash against the sector, due to the influence of illiberal ideology. This latter trend is reinforced by a global perception of decline in deservingness and public support for migrants, a sentiment that predates the Russian invasion of Ukraine [101,102].

### Major negative societal events as structure of opportunity

Research has shown that both natural and human-driven disasters can create opportunities for better developing communities and societies [2,39,103,104]. To mitigate the traumatic consequences of such crises—including wars—societies must rapidly adapt existing tools to new realities. Institutional adjustments and organizational development are likely outcomes. Following the social acceleration assumption, that societies always change in an accelerated manner [38,105] we claim that war generates a structure of opportunities for further change, especially in terms of a more flexible civic society that is able to supplement a lack of state action—this, in turn, being hampered by bureaucratic procedures. Other scholars similarly view mass refugee movements as opportunities for NGOs to counteract illiberal discourse and demonstrate resilience [106].

### The organizational and institutional ecosystem in Poland and Romania before the war

#### Migration.

In both Poland and Romania, the influx of refugees triggered a strong civic response, with ordinary citizens and NGOs providing assistance—especially at border crossings—and shaping the public sector’s response [38,107]. This was not unexpected, since, at least in some post-communist societies, NGOs have assumed the major role in terms of migrant integration over the past decade—something that reflects a lack of policy at the national and local levels [36,108,109].

In Poland, the assistance and integration system for migrants relies on short-term decisions due to the absence of a developed integration policy. The ecosystem assisting migration involves various stakeholders, but the system is not entirely open and accessible to migrants in Poland Indeed, access is contingent upon the legal status of the migrant, with two main types of actors involved: city-run institutions offering access to social services and NGOs operating within grant financing systems [110,111].

At the city level, the key institution involved in migrant integration is the social welfare center, which provides support to migrants with refugee or subsidiary protection status, including Ukrainian war refugees, who have had temporary protection status since 2022. In Warsaw, this institution is the Warsaw Family Support Center. Economic migrants with legal residence and access to the labor market are not covered but are still able to seek support from the Employment Office, another local government institution.

Local and international NGOs play a crucial role in this process, offering a range of integration activities for migrants, including legal assistance, cultural integration, psychological support, professional qualifications, language courses, and social support for those facing financial challenges. A notable and relatively recent actor is the *Konsorcjum Migracyjne* (Migration Consortium), a democratic platform for NGO cooperation.

Several NGOs operating in Warsaw have been established and are managed by immigrants, the most significant being the Ukrainian House. Notably, an increasing number of NGOs are now employing migrants as part of their workforce [112].

In Romania, the primary public institution responsible for regulating and overseeing immigration is the General Inspectorate for Immigration, which operates as a branch of the police force under the Ministry of Internal Affairs [113]. In terms of public (social) service provision, the most significant institution is the General Direction for Social Work and Child Protection. In Bucharest, the institution is subordinate to the Local Council. Another public provider of social services is the General Direction of Social Work of the City/Municipality of Bucharest, which operates under the city hall.

International organizations such as the United Nations High Commissioner for Refugees (UNHCR) and the International Organization for Migration (IOM) are active in Romania, collaborating with local NGOs to develop programs for economic migrants and asylum seekers. UNHCR’s website provides a location-based listing of organizations offering support to immigrants, covering Bucharest and other border areas. A significant stakeholder in this field is the Coalition for the Rights of Migrants and Refugees, an advocacy-focused entity comprising various NGOs. The number of NGOs in the field is, therefore, not as small as has been reported elsewhere [53], and during the initial days of the conflict, there was a sudden increase due to the involvement of other civic organizations and NGOs that were devoted to providing social services [53]. Civil society swiftly organized to address the needs of Ukrainian refugees arriving in Romania, with NGOs from diverse fields expanding their services to support them. This process involved learning and adaptation, as organizations navigated administrative and linguistic barriers [37]. These types of barriers are not new, as they have already been reported by refugees and stakeholders alike, complicating service provision and integration efforts [109].

To the best of our knowledge, there are no migrant-led grassroots organizations in Romania with a visible role in advocacy or efforts to advance discussions about the rights of migrants and refugees. This is likely due, at least in part, to Romania historically being a country of emigration with limited experience in hosting economic migrants, asylum seekers, or refugees.

#### Disability.

The disability support systems in Poland and Romania reflect characteristics typical of post-socialist disability frameworks [62,114]. Disability studies scholars have identified distinctive regional trends [58,59], particularly a tendency to portray disabled people as culturally “other” and as economic burdens on society. Social policies remain largely grounded in medical and charity models of disability. The medical model primarily views disabled individuals as patients requiring medical intervention or rehabilitation, while the charity model treats them as passive recipients of aid. While disabled people are valuable members of society and citizens of the state, neither model adequately represents them as such. Consequently, both models contribute to the segregation of disabled people into distinct impairment-related groups, failing to identify and address their needs in a holistic and intersectional manner. This approach is inconsistent with the requirements of the United Nations’ CRPD (2006), ratified by Romania in 2010 and Poland in 2012.

Persistent shortcomings in policy formulation and implementation—particularly in education, healthcare, living conditions, and social inclusion—perpetuate barriers that make it difficult for disabled people to lead independent and dignified lives [115]. Addressing these issues requires ensuring that policies and institutions are inclusive and supportive of people with disabilities. Alarming evidence points to nationwide instances where institutions intended to support disabled people have instead exacerbated their isolation and, in some cases, even exposed them to violence [60,116]. It can be argued that the primary responsibility for fulfilling the CRPD’s obligations has fallen to disability organizations in both countries rather than state authorities [60,117]. However, these organizations face significant challenges due to limited funding and resources, relying heavily on grant schemes to sustain their operations. The provision of support for disabled people is predominantly concentrated in larger urban centers in Poland and Romania.

In summary, while the impact of disability movements varies, both Poland and Romania face systemic challenges that hinder the establishment and maintenance of supportive ecosystems for individuals with disabilities. These challenges must be addressed to align national policies with the human rights and obligations outlined in the CRPD.

#### Hypotheses.

Legal provisions for disabled migrants, particularly refugees, have been part of international agreements and national regulations for several decades [35,118,119]. However, despite recognition that the intersection of refuge and disability leads to increased vulnerability, migrants with disabilities are typically marginalized both by organizations working with disabled people and those assisting immigrants and refugees [41]. This is even more salient under the adverse conditions of a residual welfare regime or under the rise of conservative stances in the society [34] Additionally, ableist practices further exacerbate exclusion [42], while the general public tends to be less sensitive to the challenges faced by individuals at the intersection of refuge and disability [43,44], an issue rooted in medieval times and persisting throughout history [45].

However, there were reported signs that change may occur when facing larger flows of incoming population displaced by war. Prior to the Ukrainian crisis, in the wake of the Syrian refugee influx, Valentinov, Bolečeková and Vaceková (40) observed that this sudden challenge helped NGOs in Austria and Slovakia clarify their identities and better structure their activities. A report on the changes in NGOs providing support to Ukrainian refugees focused on the Romanian NGOs financed by the French CARE foundation, concluding that such organizations report upscaling in terms of recruiting staff and investing in infrastructure, intensifying fundraising efforts, rethinking the structure of the organization, and providing more “tailor-made interventions” as a rule of organizational life [37]. Other reports compare NGOs to state actors, emphasizing the need for flexible interventions in the early days of conflicts [36], which demand increased speed and responsiveness [38].

Within the specific contexts of Poland and Romania, we expect similar findings and we go one step further in inspecting the consequences of these developments, particularly in terms of intersectionality and cross-sectoral cooperation. We also consider the prewar state of the sector as a key determinant of the observed impacts. When the war broke out, the efforts to provide adequate support structures for those in need were still in their initial phases in both countries. Therefore, we expect this specific intersection of refuge and disability to lead to increased vulnerability, with migration/refugee organizations and public service providers struggling to accommodate disabled refugees. Additionally, we hypothesize that the war significantly altered the daily operations of service providers—not only numerically, as scaling-up efforts were required to address the influx, but also in terms of accumulating knowledge and best practices.

Two years into the war, with no clear end in sight, practical know-how for improving existing methods likely exists “on the ground,” developed through trial and error as well as the ad hoc “good inventions” of various civil society and public actors. However, this knowledge still requires identification, formalization, and integration into broader strategies (e.g., through cross-sectoral recommendations). We also expect to observe an intensification of the cooperation across sectors; that is, between organizations supporting migrants and those working in the disability sector, with knowledge and best practices being shared across fields. In terms of the consequences for people with disabilities, we anticipate that social acceleration will have provided both the knowledge and tools for better service provision, to be used beyond situations of social emergency.

Finally, given that Polish society was already better prepared for crisis response [21], we expect the processes of change outlined above to be found to be more advanced in Warsaw than in Bucharest.

### Data and methods

In our research, we applied two distinct methodological approaches: participatory research and rapid response research. Our emphasis was on defining local priorities and perspectives integral to the participatory approach, drawing insights from the work of Cornwall and Jewkes [120]. A workshop at the beginning of the study and a group interview at the end were integral components of this approach [121]. Group interviews provided a safe space for participants to share perspectives, navigate differing opinions, and identify common ground [122], fostering a “communicative space” [121]. In addition, group interviews also ensured that the language and concepts used during discussions are rooted in participants’ experiences [123]. The first group interview helped shape the research design and recruitment process, while the second allowed participants to engage with preliminary findings and suggest practical solutions. This iterative approach enabled the researchers to incorporate participant feedback during both the design and interpretation phases.

Simultaneously, the rapid response research approach aimed to swiftly develop responses to emerging problems, following the framework proposed by Stimson, Fitch [124]. Ethical considerations, as highlighted by [125], and recommendations in Poland [126], led us to focus on helpers, not on refugees. Purposive sampling guided stakeholder selection based on two criteria: experience in supporting migrants/refugees and experience in supporting people with disabilities. To ensure balanced representation, we carefully controlled the representation of public and non-public actors. The research was approved by the Research Ethics Committee at Collegium Civitas (7/7/2023).

Fieldwork was conducted between July 15 and December 15, 2023, at two research sites: Warsaw and Bucharest. All the participants provided oral informed consent before the interview, after receiving explanations about the research study’s purpose, anonymity measures, and their right to withdraw from the interactions with the researchers at any time. Consent was reiterated, recorded, and transcribed at the start of each interview. The consents are documented on both recordings of the interviews and the transcripts. The Research Ethics Committee at Collegium Civitas requires written consent only when interviewees receive remuneration; therefore, oral consent was an approved procedure. This approach aimed to minimize potential respondent reluctance and streamline the interaction process, particularly for online interviews.

The research unfolded in three stages: preliminary research, involving an initial workshop with stakeholders supporting either migrants/refugees or people with disabilities; individual in-depth interviews (41); and an extended group interview discussing hypothetical cases of refugees with disabilities. The fieldwork conducted at the two sites followed the same approach. Additional information was collected during research visits to service providers in Bucharest (3) and Warsaw (3).

The initial workshop was particularly valuable for establishing connections, building a potential database of respondents, and gaining preliminary insights into the day-to-day challenges of professionals. It also helped refine the research instruments and adjust the list of respondents.

The most challenging aspect of data collection was conducting in-depth interviews with individual stakeholders. Scheduling conflicts, fatigue, and overwork posed difficulties. In Romania, the limited number of relevant cases further complicated participant recruitment. In Poland, the delay was exacerbated by parliamentary elections, which caused some public officials to be reluctant to participate.

Table 1 depicts the resulting sample. Most interviews were one on one, although a few involved two or three respondents. The specificity of the research theme likely deterred participants, as many had limited experience with cases of refugees with disabilities. Additionally, the participation of multiple respondents in the same interview was perceived as an attempt to maximize the researchers’ gains or assist them in gathering more relevant information.

**Table 1:** Structure of the sample.

	Bucharest		Warsaw	
	No. of interviews	No. of interviewees	No. of interviews	No. of interviewees
Representatives of local authorities (providers of public services)	6	8	2	2
Representatives of NGOs in the field of disabilities	4	4	9	9
Representatives of NGOs* in the field of refugees/immigration	5	5	5	5
Representatives of NGOs/stakeholders extending their services to refugees from Ukraine	3	4	1	1
Volunteers involved in offering help to Ukrainian refugees	2	2	1	1
Representatives of public institutions (above local authorities)	0	0	3	3

*Including one academic in Bucharest, but also NGO member.

The response rate was relatively low at both research sites. In Bucharest, 20 interviews were completed after reaching out to 60 individuals, while in Warsaw, 21 were conducted after approaching nearly 50 potential respondents. Interviews were conducted face to face (6 in Romania, 9 in Poland), online (12 in Romania, 8 in Poland), or by phone (2 in Romania, 4 in Poland), depending on the respondents’ preferences.

After completing at least 20 interviews, we organized an extended group interview with relevant stakeholders, moderated by an external expert. This workshop focused on discussing potential cases of refugees with disabilities as well as addressing issues such as available resources, access, and possible setbacks. The researchers defined the cases, which were then provided to the participants and the moderator. This event served to validate the research findings and enrich them with practical insights from local actors. Its added value stemmed from bringing together stakeholders with diverse backgrounds—NGOs (in Poland and Romania), public administration (in Poland and Romania), and academia (in Romania)—while fostering collaboration and consensus on the cases discussed.

At each stage of the research, respondents were informed about the study’s purpose and its sources of funding. Each participant provided consent for the interviews and recordings, and all interviews were anonymized to ensure confidentiality. The transcripts were translated into English, and the authors read and coded the interviews using Atlas.TI, employing a coding system with 68 entries. Each interview was coded by two researchers, and intercoder reliability was tested, along with the reliability for each code. All codes proved to be reliable, and interpretation was based on them, leading to the findings presented in the next section.

## Findings

### Strategies adopted by service providers

#### Warsaw.

Both NGOs and public service providers were unprepared to help assist the large influx of refugees, including those with disabilities. The initial response was chaotic but highly engaged, with grassroots organizations playing a key role. Public institutions primarily operated within the legal frameworks that defined their target groups and methods of assistance. When possible, public agencies and NGOs adopted inclusive approaches: citizenship was not a criterion for aid as long as beneficiaries legally resided in Poland. However, an exception applied to state social security support for individuals with disabilities, which required a Polish disability certificate. In other instances, a self-declaration of disability sufficed. State agencies did not provide direct assistance but instead transferred money to other entities, such as NGOs or local governments, to organize support for UWRwD:

*(Anonymized) directly does not provide this support in the sense of organizing accommodation, meals, or rehabilitation activities for individuals from Ukraine. Instead, (anonymized) outsources these services to other entities.* (PL19, M, agency of public administration)

Building networks of cooperation was a common strategy for local government agencies and NGOs. No single organization provided comprehensive support; instead, they focused on specific areas and redirected beneficiaries to others when additional assistance was needed. For example, one disability-focused organization (PL06) cooperated with a migration organization when a translation was needed, while another provided food and yet another offered financial support. In turn, the disability-focused organization assisted others by supplying equipment for people with physical disabilities:

*Of course [we have allies], because they also work towards our goals, and that’s why very often such situations where we can recommend our Foundation to people, like recommending us from other foundations, we do the same.* (PL06, F, NGO focused on persons with disabilities)

However, cooperation between the non-governmental and public sectors was limited. NGOs primarily interacted with state institutions through grant applications. Many NGOs perceived public institutions as only temporarily engaged and slow to organize aid. Indeed, after some time, public institutions were observed to be withdrawing from assistance efforts (GI).

Local government, to some extent, and NGOs based their systems of helping Ukrainian war refugees with disabilities on the voluntary work of both Poles as well as Ukrainians. This was particularly true during the first few months of the crisis, when the flow of refugees outpaced available human resources, experience, and procedures:

*We ask the refugees themselves who stay in the hostel to take care of such a person in these basic matters […] Sometimes we have friends who do this work […] but it’s not a systemic solution.* (PL04, M, NGO focused on immigrants)

Over time, with increased funding, NGOs started to hire new employees and, in some cases, opening additional offices.

It seems that the main differences in approach to helping Ukrainian war refugees with disabilities are not between public and non-governmental entities, but rather between those state entities that support refugees directly and those that support them indirectly. Direct interaction with beneficiaries fostered shared experiences among aid providers. However, these experiences did not translate into new procedures or regulations for future refugee crises in Poland.

#### Bucharest.

According to our interviewees, Romania experienced a similar flow of refugees, marked by surprise, a lack of preparedness, and chaotic initial responses. Local NGOs, being more flexible in their organization, played a central role in migration-related efforts, with those specializing in social services intervening and providing unstructured help. Even after state agencies established refugee camps, NGOs—now supported by their transnational branches—remained essential, particularly in tailoring interventions to refugees’ specific needs, including UWRwD. However, neither the NGO sector nor state agencies had dedicated provisions for refugees with disabilities. Intersectionality became an increasingly pressing issue, not only concerning the recognition of disability certificates but also in realizing the broader gaps in service provision:

*We are deficient in providing services to people with disabilities, of any kind of disability. People with physical disabilities are more visible, obviously. But we are still far from providing any services for people with disabilities.* (RO17, F, NGO focused on other issues than refugees or disabled)

Cross-domain cooperation between migration and disability sectors was driven by the particular challenges encountered, primarily in terms of the capacity to support refugees. NGOs focused on immigration-related issues, including refuge, increasingly referred UWRwD to NGOs in the social sector, especially those dealing with disabilities. At least until mid-2023, the problem was not financing but rather human resources, provider burnout, skill shortages, the availability of appropriate tools and facilities, and advocacy capacity. The social acceleration assumption proved accurate, as one interviewee pointed out:

*Cooperation was due to overcrowding; that is the main dominant criterion. Access to funds was secondary because, in general, since the war started and until the end of this year, there have been more funds than the capacity to help […] At least that’s how I estimate it. That is, no one complained about... about a lack of... of funds. On the contrary, there were organizations that said, “oh, too many donors come to us”; there were organizations that had to give up some donors by directing them to others.* (RO14, M, NGO focused on migration issues)

Public agencies developed creative solutions to address both regulatory and funding gaps, including using personal resources, improvising, and allowing NGOs to provide services within public agency spaces. Again, cooperation arose primarily from the need to deliver services in response to overcrowding. Since this need was evident early on, public providers were receptive to NGO assistance from the outset.

Both state agencies and NGOs reported that legislation was ancillary, slow to evolve, and lacked an adequate understanding of the actual needs of refugees, particularly those with disabilities. One interviewee stated this explicitly:

*If the Romanian state has decided to do this, to make this change, it did a good thing. In my opinion, it should have been done a long time ago because they got used to the idea that we have rights for too long. And suddenly, on May 1st, the state came with the ordinance [...] you must integrate. In my opinion, this should have come gradually, it was a year to stay, you have the right, receive everything, and then […] they were pushed, as they say, onto the streets, alone. It was all of a sudden, that’s my opinion. They are still people who need help, they are still disappointed, disillusioned, frightened, worried. Yes, the change came too abruptly. I believe this change should have come after 3, 4, 5, 6 months.* (RO09, F, public refugee center)

Intersectionality was only considered in the later phases of the refugee response, coinciding with the emergence of cross-domain cooperation. This cooperation was not programmatic but reactive, driven by the need to address immediate challenges. NGOs came forward to help public service providers, and migration-focused NGOs became more open toward cooperating with disability-focused organizations. While there was little transfer of knowledge and best practices between domains, we encountered accounts of intra-domain cooperation with service providers primarily working with others in their own field.

### Changes observed among service providers

#### Warsaw.

The first change for service providers was the emergence of a new type of beneficiary:

*Interviewer: Regarding war refugees with disabilities, how long has your organization been involved in this issue?*

*Respondent: For us, the moment of the outbreak of the war in Ukraine, the Russian invasion, was when we started thinking about such issues.* (PL15, F, NGO focused on disability)

This shift led to other changes, prompting NGOs to adopt new strategies in response to evolving demands. Disability- and migration-focused NGOs leveraged existing support networks to enhance their efforts and formed new alliances to extend their capacities:

*I saw a great need to be better prepared both in terms of expertise and, I mean, practically. I mean, I’m talking about the expertise that can be applied, right? […] I think it was a need for better information flow between organizations themselves. But we tried to fix it somehow by creating these shared files, whether on drives or creating this communication channel. We tried to somehow fix it and meet that need.* (PL15, F, NGO focused on disability)

Using networks provided opportunities for acquiring knowledge and solutions

*And, honestly, I don’t know if we would have taken it on in such an organized way […] if it weren’t for the support from organizations that are more experienced in this field.* (PL15, F, NGO focused on disability)

Another key adaptation was the expansion of human resources. NGOs hired additional staff, many of whom were bilingual or multilingual, to help overcome language barriers between service providers and refugees:

*I think here many organizations also experienced this sudden growth, and a lot of new people were hired, who often had to be trained because, as we mentioned, bilingual individuals were in high demand, and many of them, like me, completely changed their profession. So, it was necessary to quickly acquire a lot of knowledge.* (PL02, F, NGO focused on immigrants)

To meet the diverse needs of refugees, NGOs developed new services and projects, such as bilingual hotlines, video services for the deaf, accessible information centers, and customized support services. This expansion required a strengthening of administrative functions to ensure effective operations and regulatory compliance:

*Earlier, we never dreamed of such, such, such large amounts of money in the account, so to speak. Such large funding, it sounded nice. And that was such a leap, both financially and in terms of staff; our team expanded, and our experience as well. So, we had to learn a lot and gain a lot of experience. When I opened the point, which was again a new place, in a new location, something completely new, I approached it differently, also more calmly.* (PL07, F, NGO focused on disability)

Employees and volunteers gained new skills, particularly in managing sensitive and complex situations related to the refugee crisis:

*Not only did we need knowledge and access to information to provide people with reliable, good, and up-to-date information but also psychologically, it was challenging. We learned a lot in those first months, but we also understood that the knowledge and so-called hard skills we have need improvement. Also, as employees on the first line, we need improvement in the emotional sense to take care of ourselves, our rest, and our overall well-being. Improving qualifications in working with people in crisis is essential.* (PL02, F, Polish NGO focused on assisting Ukrainians)

A public service provider in Warsaw also experienced a significant expansion in human resources and the type and number of beneficiaries, although the latter has since decreased:

*After February 24th, [anonymized] expanded by nearly a hundred employees due to the task assigned by the Voivode, involving long-term stay points for refugees from Ukraine. Initially, there were nine points, and currently, we have five, accommodating a total of approximately 1,020 individuals, give or take.* (PL13, F, agency of public administration)

Service providers that did not have direct contact with UWRwD created new programs and grant opportunities for NGOs directly assisting them. They also established funds but did not experience an increase in human resources.

Overall, service providers experienced significant changes in the type and scope of their activities as well as in their workforce. They gained extensive knowledge and experience in assisting refugees with disabilities. However, these changes have been dynamic, influenced by the declining number of UWRwD and decreasing support from both public institutions and society.

#### Bucharest.

Adaptability and upscaling were fundamental to adequately responding to the needs of Ukrainian refugees, who represented a new category of beneficiaries. For NGOs in the field of immigration, the novelty resided in the country of origin, while for disability-focused NGOs, the beneficiaries’ status introduced new complexities due to their multilayered vulnerability as both displaced and disabled individuals. Both public and civil-society-based actors played a role in providing services to Ukrainian refugees with disabilities, and most often communication between the various entities proved to be key when dealing with the complex needs that emerged. The cooperation between public providers/structures and NGOs was an effective way of making sure that potential beneficiaries could access the services they required in time, due to the flexibility of the latter. However, directing beneficiaries with disabilities to appropriate service providers was not always straightforward and involved various challenges:

*The main difficulty is to find organizations that deal with disabilities, except for the parts of autism, where there are organizations that respond.* (RO12, F, NGO focused on other issues than refugees or disabled)

Cooperation was part of the learning process, especially for NGOs that had not previously worked in immigration or refugee aid but had to quickly adapt to the evolving situation. Initially, NGO activities were guided by immediate needs encountered in the field. Over time, however, they became more structured and coherent organizations gained experience in handling specific cases. Specific types of problems were encountered, and cooperation played a significant part in dealing with them, alongside constant communication with the beneficiaries:

*With time, the organizations’ actions became more structured. We identified the needs and aligned ourselves more effectively. However, at the beginning, it was more of an improvisational effort […] We couldn’t predict the problems that would arise the following week […] In other words, after offering assistance, we need to get feedback to see if we did well or if we need to adjust something […] I thought that there were some lessons we learned from there, and we learned to see them. They are somehow knowledge, experiences that our international partners, who usually do this, brought to us.* (RO03, F, NGO focused on other issues than refugees or disabled)

For public service providers, the sharp increase in the number of beneficiaries was not accompanied by a proportional increase in the number of employees, often resulting in overwork. In contrast, NGOs experienced organizational growth as their pool of potential beneficiaries expanded. This expansion was closely guided by the needs encountered “on the ground,” and, at least initially, involved a significant amount of learning by doing. The existence of various funding schemes facilitated the upscaling process, enabling NGOs to expand their teams with case workers and administrative personnel to better pursue financing alternatives. However, significant challenges arose in recruiting translators, and as many NGOs broadened their scope, finding the most suitable professionals became increasingly difficult due to the high demand for skilled personnel. Meanwhile, public service providers were often assigned to handle Ukrainian refugees without adequate preparation, leading to unsustainable workloads:

*We came here for the first time, we got to know each other. In the meantime, we changed many people in the team because some didn’t want to, some couldn’t cope, some had various problems. We changed people; we weren’t a constant team.* (RO011, F, agency of public administration)

However, what started as exponential growth stopped over time, due to the diminishing of the available funds, leading to insecurities and uncertainties regarding the sustainability of some of the services that were provided and, accordingly, the sustainability of maintaining the newly hired professionals. In this context, thorough documentation of case handling and good practices would probably have been an effective organizational tool for enhancing knowledge retention, beyond economic and structural fluctuations.

## Conclusion and discussion

Our research provides a cross-sectional analysis of experiences of helping refugees with disabilities in the face of negative events. These situations require quick action but, at the same time, create structures of opportunity. The study examined how and to what extent these structures of opportunity were leveraged, introducing an intersectional perspective to the discourse on supporting refugees with disabilities.

We focused on two countries with limited prior experience in assisting refugees, particularly those with disabilities. Both are post-communist states with relatively weak civil societies and ongoing struggles with the illiberal wave. The findings may be generalized to other societies with similar histories and experiences regarding (not) helping refugees with disabilities.

We argue that key institutional changes occurred in three main areas: material and human resources, cooperation networks, and programmatic upscaling. While both NGOs and public agencies experienced these shifts, upscaling was more pronounced among NGOs. In response to sudden adverse events, a learning process emerged, enabling service providers to adapt their organizational structures and tailor their services to better meet the needs of their beneficiaries. This process helped counteract the compounded vulnerabilities experienced by disabled refugees, as observed elsewhere [127].

Regarding material and human resource upscaling, the most significant factor was the unprecedented financial support received by NGOs, particularly those migration- and disability-focused organizations in Poland and migration-focused organizations in Romania. This funding allowed NGOs to strengthen their human resources by hiring more qualified staff, including administrative personnel and case workers with expertise in migration. Expansion enabled these organizations to provide a broader range of assistance and acquire additional physical space to accommodate more people in need. In Poland, some organizations even opened new branches, while in Romania, NGOs often operated at the borders with Ukraine and Moldova, providing on-site assistance—an entirely new experience for many.

In contrast, public providers experienced limited upscaling, with the exception of one agency in Warsaw. Bureaucratic constraints and a more rigid operational structure prevented significant expansion. However, public agencies did take on new responsibilities, such as distributing public funds to NGOs and organizing reception centers, thereby broadening their scope even though their overall capacity did not expand as substantially as that of NGOs.

Organizations helping disabled refugees had to expand their missions, primarily through hands-on learning. Many found themselves unprepared to address the multifaceted needs arising from displacement, disability, age, and other characteristics of refugees. This led to an evolving collaboration between disability-focused and migration-focused NGOs in Poland and Romania, although the nature of this collaboration varied between the two countries. In Poland, both types of NGOs actively built networks and exchanged information, whereas in Romania, migration-focused organizations demonstrated more cross-sectoral initiative than disability-focused organizations.

As a result, intersectionality was more present in Poland’s interventions. As argued, a slightly more developed welfare provision is in place there as compared to Romania, which is likely to constitute the premises for integrating innovative ways of helping disabled refugees. Otherwise, the typical absence of disability-specific provisions in immigration policies [24], as well as inconsistencies between the biomechanical definitions used by health systems and the more nuanced definitions applied by other service providers [23,24], were evident in both capitals under scrutiny. However, the urgency of immediate intervention accelerated a harmonization process—at least at the level of practice, if not policy and regulation.

The organizations acquired knowledge on fundraising and managing large new projects. Through contact with a new type of beneficiary, they gathered information about and built expertise in supporting refugees with disabilities. In the case of UWRwD, neither legal residence nor access to public services was a primary issue, but we observed that support providers had to learn how to assist UWRwD with accessibility and language barriers as well as approaches to defining, diagnosing, and treating various disabilities. Gathering this knowledge was one of the most important dimensions of the organizations’ upscaling. However, most organizations have not successfully transformed this accumulated practical knowledge into more established solutions, such as written procedures. This pattern confirms the existing trend among civil society organizations. Due to a lack of funding for long-term institutional development, these organizations often lack have the capacity to translate their acquired knowledge into structured approaches or systematic solutions. Such solutions, if integrated into institutional knowledge, could be transferable across organizations and would not rely solely on the people who originally developed them, ensuring that they remain within the organizations over time.

At the beginning of our research, we distinguished between two categories of service providers—public agencies and NGOs—to analyze their experiences in supporting UWRwD. Our findings revealed that public agencies were generally slower and less flexible in assisting refugees with disabilities. However, we observed differences between public agencies with direct versus indirect contact with UWRwD. The former, primarily local government actors with frontline workers engaging directly with UWRwD, had experiences more akin to those of NGOs, as they were often forced to implement non-standard solutions. Consequently, they occasionally reached out to NGOs, fostering cross-sectional support networks. These public agencies also experienced knowledge growth in assisting refugees with disabilities, a direct result of their engagement. However, as with NGOs, this knowledge was not systematically codified.

Analysis of the collected data revealed patterns of organizational growth in both human resources and programmatic growth across the two countries. In the initial phase immediately after the outbreak of the war, NGOs—despite limited resources and a lack of experience—began to support refugees with disabilities almost immediately. This led to overwork and burnout among staff. The subsequent influx of funding allowed for the hiring of additional personnel, stabilizing operations and enabling expansion. Public agencies only entered the field after legal frameworks were established to facilitate their involvement. Despite this, the primary burden of assistance remained with NGOs.

In terms of programmatic assistance, the early phase followed a recognizable: identifying the needs of refugees, assessing internal capabilities, devising solutions, providing assistance, or redirecting beneficiaries to other organizations. This approach to the development of a support network among NGOs, enhanced service provision and allowed for more responsive and effective aid. As organizations gained experience, the process of helping became smoother. Once the needs of UWRwD were diagnosed, NGOs typically knew where to direct individuals. Continuous engagement with new cases provided insights into the best ways of helping refugees and navigating multicultural perceptions of disability.

This pattern was observed in both Warsaw and Bucharest, although with notable differences. Cross-sector cooperation was limited overall but appeared to be stronger in Bucharest than in Warsaw, whereas within-domain cooperation was more common in Warsaw than in Bucharest. This contrast likely stems from the greater involvement of local authorities in organizing help for UWRwD in Bucharest, whereas in Warsaw, the NGO sector supporting people with disabilities was more visible and involved in providing services to Ukrainian refugees with disabilities in Warsaw than in Bucharest.

From a paradigmatic viewpoint, intersectionality proved to be a valuable framework for analyzing these processes. Beyond the individual-level intersection of disability and refugee status, our research also highlighted variations shaped by societal contexts. Structural power dynamics and intergroup relations emerged as additional layers in the intersections. While these aspects were only marginally addressed in our findings due to the study’s scope, we recommend them as areas for further reflection and research.

Our research also has a series of limitations. Because we interviewed organizations in Warsaw and Bucharest, our findings may be generalizable to other large cities, but they do not capture the experiences of assistance in border areas or smaller towns. Another limitation is the exclusion of refugees with disabilities and their caretakers, whose daily caregiving experiences were not directly examined. Future research should prioritize gathering and analyzing the experiences of refugees with disabilities themselves. Additionally, further investigation is needed to assess whether and how cross-sectional cooperation is maintained within the evolving national contexts of Poland and Romania and across the broader geopolitical landscape. Another relevant field of study is the evolution of the intersectionality discourse in Poland, Romania, and other Central and Eastern European countries, particularly concerning disability and refugee status.

Overall, while this “unfortunate natural experiment” facilitated capacity building—particularly among NGOs—and fostered previously uncharted networking and cross-sector cooperation between NGOs and public service providers in Poland and Romania, these upscaling trends could be unstable or reversible. This instability may depend not only on available funding but also on the human capacity to sustain work in highly stressful conditions marked by constant change and uncertainty, even among service providers strongly motivated to help people in need.

As for policy recommendations, the key priorities include developing long-term policies to support refugees with disabilities and ensuring their inclusion in relevant laws and programs at both the national and local levels.
